# Myocardial involvement characteristics by cardiac MR imaging in neurological and non-neurological Wilson disease patients

**DOI:** 10.1186/s13244-023-01583-7

**Published:** 2024-01-25

**Authors:** Wei Deng, Jie Zhang, Zhuoran Jia, Zixiang Pan, Zhen Wang, Huimin Xu, Liang Zhong, Yongqiang Yu, Ren Zhao, Xiaohu Li

**Affiliations:** 1 Department of Radiology, Research Center of Clinical Medical Imaging, Anhui Province Clinical Image Quality Control Center, The First Affiliated Hospital of Anhui Medical University, No. 218 Jixi Road, Hefei, 230022 China; 2 Department of Neurology, Institute of Neurology, Anhui University of Traditional Chinese Medicine, Hefei, China; 3 Department of Cardiology, The First Affiliated Hospital of Anhui Medical University, No. 218 Jixi Road, Hefei, 230022 China; 4 Duke NUS Medical School, National Heart Centre Singapore, National University of Singapore, Singapore, Singapore

**Keywords:** Wilson disease, Copper, Myocardial fibrosis, Cardiac magnetic resonance

## Abstract

**Objectives:**

To explore the characteristics of myocardial involvement in Wilson Disease (WD) patients by cardiac magnetic resonance (CMR).

**Methods:**

We prospectively included WD patients and age- and sex-matched healthy population. We applied CMR to analyze cardiac function, strain, T1 maps, T2 maps, extracellular volume fraction (ECV) maps, and LGE images. Subgroup analyzes were performed for patients with WD with predominantly neurologic manifestations (WD‐neuro +) or only hepatic manifestations (WD‐neuro −).

**Results:**

Forty-one WD patients (age 27.9 ± 8.0 years) and 40 healthy controls (age 25.4 ± 2.9 years) were included in this study. Compared to controls, the T1, T2, and ECV values were significantly increased in the WD group (T1 1085.1 ± 39.1 vs. 1046.5 ± 33.1 ms, T2 54.2 ± 3.3 ms vs. 51.5 ± 2.6 ms, ECV 31.8 ± 3.6% vs. 24.3 ± 3.7%) (all *p* < 0.001). LGE analysis revealed that LGE in WD patients was predominantly localized to the right ventricular insertion point and interventricular septum. Furthermore, the WD‐neuro + group showed more severe myocardial damage compared to WD‐neuro − group. The Unified Wilson Disease Rating Scale score was significantly correlated with ECV (Pearson’s *r* = 0.64, *p* < 0.001).

**Conclusions:**

CMR could detect early myocardial involvement in WD patients without overt cardiac function dysfunction. Furthermore, characteristics of myocardial involvement were different between WD‐neuro + and WD‐neuro − , and myocardial involvement might be more severe in WD‐neuro + patients.

**Critical relevance statement:**

Cardiac magnetic resonance enables early detection of myocardial involvement in Wilson disease patients, contributing to the understanding of distinct myocardial characteristics in different subgroups and potentially aiding in the assessment of disease severity.

**Key points:**

• CMR detects WD myocardial involvement with increased T1, T2, ECV.

• WD‐neuro + patients show more severe myocardial damage and correlation with ECV.

• Differences of myocardial characteristics exist between WD‐neuro + and WD‐neuro − patients.

**Graphical Abstract:**

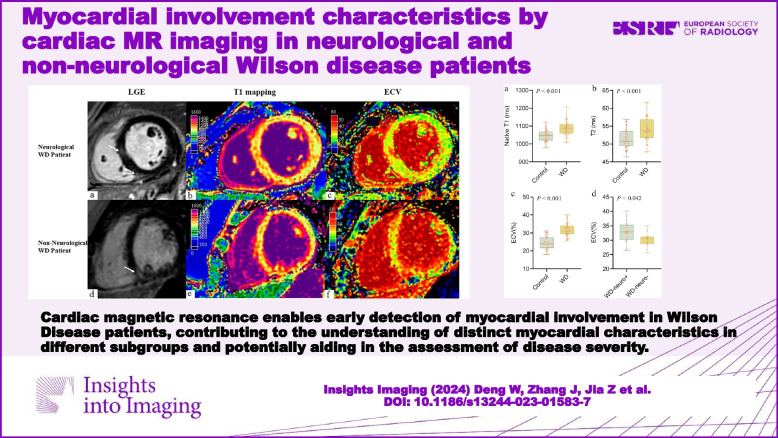

## Introduction

Wilson disease (WD) is a genetic disorder of copper metabolism caused by variants in the ATP7B gene [1, 2]. Globally, the prevalence of the disease gene is 1 in every 90 individuals, and the occurrence of the disease ranges from approximately 0.25/10,000 to 4/10,000 [3, 4]. Copper overload mainly occurs in the liver and brain and causes a variety of liver damage and neurological symptoms. Patients can be divided into WD-neuro + (patients with at least one neurological symptom) and WD-neuro − (patients with a primarily hepatic involvement) patients [5, 6]. However, several studies have shown that copper deposition can also occur in the heart and lead to clinical symptoms of related cardiovascular disease (e.g. arrhythmias, atrial fibrillation, heart failure, etc.) [6–13]. Therefore, early detection of myocardial involvement is important even in asymptomatic WD patients. Electrocardiogram and echocardiography are the most commonly used cardiac examination to evaluate WD [14–17]. However, their ability to detect alteration in myocardial microstructure and function is limited, and the absence of distinctive characteristics poses a challenge in recognizing myocardial damage caused by WD. [13, 18]. Endomyocardial biopsy is the gold standard for the diagnosis of many primary and secondary cardiac conditions [19]. Unfortunately, it is difficult to be widely carried out in clinical work because it is an invasive method and has the risk of complications. Cardiac magnetic resonance (CMR) imaging has become a non-invasive and reliable tool for detecting and diagnosing myocardial involvement in a variety of diseases [20, 21]. CMR provides comprehensive evaluation of cardiac morphology, function, and myocardial fibrosis by CMR-T1 mapping, T2 mapping, and late gadolinium enhancement (LGE) [7, 22, 23]. Previous study [6] had shown that in German cohorts, WD-neuro + patients exhibit more adverse cardiac remodeling and dysfunction compared to WD-neuro − patients. However, the characteristics of myocardial involvement in WD patients among Asian populations remain unclear.

We aimed (1) to evaluate the segmental involvement characteristics of myocardium in WD patients using CMR and (2) to compare the differences between WD-neuro + and WD-neuro − patients.

## Material and methods

### Patients

From April 2022 to November 2022, a prospective and consecutive recruitment of 48 patients newly diagnosed with WD was conducted at First Affiliated Hospital of Anhui Medical University. The inclusion criteria included (1) patients diagnosed with WD according to the WD diagnostic guidelines published by the European Association for the Study of the Liver [24] and (2) patients agreed to CMR examination. The exclusion criteria included (1) contraindications of MRI (e.g., implanted metallic objects, claustrophobia, etc.); (2) patients had a history of cardiac disease; and (3) patients had other cardiovascular risk diseases factors (e.g., hypertension, diabetes, etc.). The patient's clinical history, laboratory tests, electrocardiogram, and echocardiogram results were acquired. The WD patients were divided into two groups: WD-neuro − and WD-neuro + group [6]. We calculated the Unified Wilson Disease Rating Scale (UWDRS), which describes neurologic signs and their severity [25]. In addition, we recruited volunteers through advertisement and invitations. All the controls had no known risk factors for cardiovascular disease, a family history of heart disease, previous hospitalization, and were not taking any cardiovascular medications. The study was approved by the hospital Ethics Committee. Written informed consent was obtained from all subjects (Ethics number: PJ2022-09–59).

### CMR imaging protocol

CMR was performed on a 1.5 T whole-body MR system (Ingenia, Philips Healthcare, Best, The Netherlands). Patients were scanned using a 32-element body array coil. A stack of contiguous parallel short-axis views covering the entire left ventricle (LV) and right ventricle (RV) from base to apex and three LV long-axis views (two-, three-, and four-chamber). CMR-cine was performed using a balanced steady-state free precession sequence with breath holding, acquiring 30 phases. Native T1 mapping and postcontrast T1 mapping were performed at the basal, middle, and apical levels of the LV short-axis using a modified Look-Locker Inversion recovery (MOLLI, 5(3)3 protocol) and MOLLI (4(1)3(1)2) protocol) sequence, respectively. T2 mapping was performed using a multi-echo gradient-spin-echo sequence, and the slice location was the same as T1 mapping. LGE imaging was performed using a segmented phase-sensitive inversion recovery sequence within 10 to 15 min after injection of gadolinium contrast agent (0.2 mmol/kg) using the same views as cine images. The details of the CMR imaging acquisition parameters are summarized in Table 1.

**Table 1:** MR imaging parameters

Parameters	Cine	T1 mapping	T2 mapping	LGE
FOV (mm^2^)	300 × 300	300 × 300	300 × 300	300 × 300
Slice thickness (mm)	8	7	10	6
Acquired pixel size (mm^2^)	1.97 × 2.26	2.0 × 2.0	2 × 2	1.6 × 1.96
Recon voxel size (mm^2^)	1.04 × 1.04	1.17 × 1.17	1.04 × 1.04	0.89 × 0.89
Matrix size	152 × 133	152 × 150	152 × 148	188 × 153
SENSE	2	2	3	2
TR (msec)/TE (msec)	3.4/1.52	3.0/1.40	750/9.3(∆TE)	6.0/3.0
Flip angle (°)	60	35	90	25

*FOV* field of view, *LGE* late gadolinium enhancement

### Post-processing and analysis

The post-processing of all CMR images was performed by two radiologists (W.D. and Z.P., both with 2 years’ experience in the evaluation of CMR) using Cvi42 (Version 5.6.6, Circle Cardiovascular Imaging Inc., Calgary, Canada). Any differences between the two observers were adjudicated by a senior observer (X.L., with 15 years’ experience in CMR). Automated delineation of the LV endocardial, epicardial, and left atrial (LA) endocardial contours (excluding papillary muscle, pulmonary veins, and LA appendages) was conducted at both systole and diastole endpoints within the cine images (with manual adjustment if necessary). Subsequently, automatic calculations were executed, leading to the generation of cardiac functional parameters. CMR two-dimensional global peak left ventricle strain from feature tracking was assessed as previously described [26]. The native T1 maps, postcontrast T1 maps, and T2 maps were automatically generated by importing the original native T1 images and postcontrast T1 images into the corresponding analysis module. Venous blood samples were taken within 24 h before CMR examination, and the hematocrit level of all subjects was measured. The CMR-derived extracellular volume fraction (ECV) was calculated using the methods described previously [27]. The calculation formula was as follows:

$${\text{ECV}}=(\Delta {\text{R}}1{\text{myo}}/\Delta {\text{R}}1{\text{blood}})\times (1-{\text{hematocrit}})$$

Among them, ΔR1myo is the difference between the T1 value of myocardial tissue and the T1 value of blood, and ΔR1blood is the change in the T1 value of blood. Native T1 maps, T2 maps, and ECV maps were analyzed using the 16 segments model using the classic AHA guide [28] of the LV (excluding the apical segment). At our center, we established the upper normal limits for ECV, native T1, and T2 values at the 95th percentile, with threshold of 29% for ECV, 1090 ms for native T1, and 56 ms for T2. The segments were defined as having myocardial involvement if there were prolonged ECV, native T1, and T2 in any segment. The same two radiologists reviewed the LGE images. The location and pattern of LGE lesions were evaluated by manually adjusting the endocardial and epicardial contours that automatically delineate the myocardium on LGE images. LGE lesions were defined as signal intensity greater than 5 standard deviations above the remote reference myocardial average signal intensity.

Intra-observer reliability was assessed from repeated measurements made by one radiologist blinded to previous results of 20 random subjects obtained at least 1 week later. Inter-observer reliability was independently assessed on the data of 20 subjects by another radiologist blinded to the first radiologist's measurements.

### Statistical analysis

Statistical analysis was performed using SPSS (version 26.0, Statistical Package for the Social Sciences, International Business Machines, Inc., Armonk, NY, USA). Data were checked for normal distribution using the Shapiro–Wilk test. Continuous and normally distributed variables (Kolmogorov–Smirnov test, *p* ≥ 0.05) were expressed as mean ± standard deviation. Differences between continuous variables were tested using the Student’s *t*-test. Categorical variables were expressed as *n* and percentage, Differences between categorical variables were tested using Fisher's exact test. Multivariate analysis was performed using multiple linear regression. The Pearson correlation coefficient (*r*) was used to assess linear relationships between variables. After utilizing Benjamini–Hochberg procedure, the false discovery rate-corrected *p*-values lower than 0.05 were considered statistically significant.

## Results

### Baseline characteristics

**Fig. 1:**
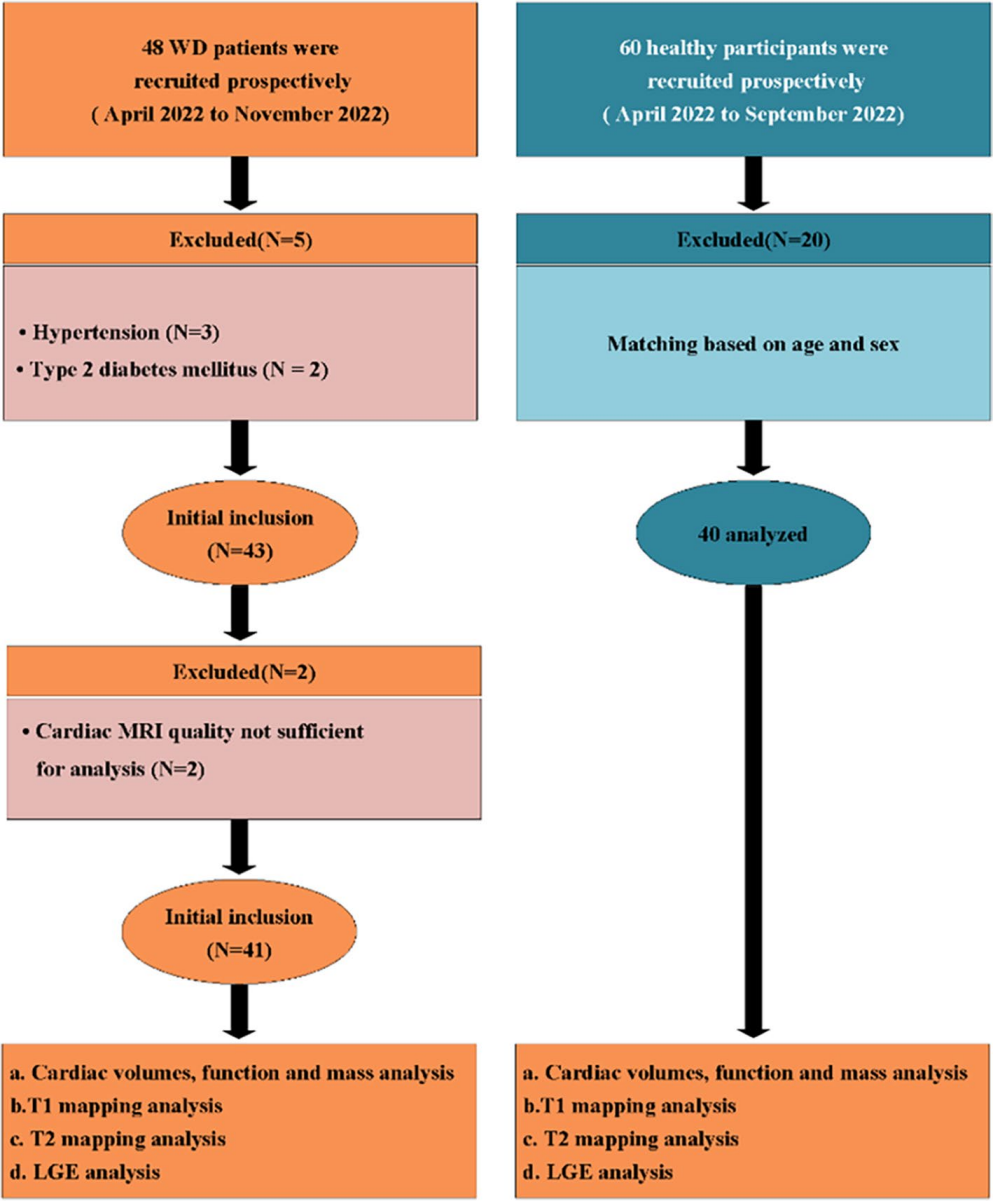
Flowchart of study participants

The baseline characteristics of the study population are presented in Table 2. A total of 48 patients were enrolled, and 7 patients were excluded for poor image quality (*n* = 2), hypertension (*n* = 3), and type 2 diabetes mellitus (*n* = 2). Finally, 41 WD patients (age 27.9 ± 8.0 years, 59% men) and 40 healthy controls (age 25.4 ± 2.9 years, 58% men) were included in this study (Fig. 1). No echocardiographic abnormalities were found in any of the patients. ECG abnormalities were found in 19 patients, including premature ventricular contractions (*n* = 4), sinus bradycardia (*n* = 11), and sinus arrhythmia (*n* = 4). The median UWDRS score of WD-neuro + patients was 6.0 (1–11).

**Table 2:** Baseline characteristics for WD patients and controls

Characteristics	Controls (*n* = 40)	WD (*n* = 41)	*p*	WD-neuro − (*n* = 17)	WD-neuro + (*n* = 24)	*p*
Age (years)	25.4 ± 2.9	27.9 ± 8.0	0.189	24.2 ± 5.6	30.5 ± 8.5	0.149
Gender			0.991			0.371
Men, *n* (%)	23 (58%)	24 (59%)		7 (41%)	17 (71%)	
Women, *n* (%)	17 (42%)	17 (41%)		10 (59%)	7 (29%)	
BSA (m^2^)	1.7 ± 0.2	1.7 ± 0.2	0.717	1.7 ± 0.3	1.8 ± 0.2	0.805
BMI (kg/m^2^)	22.0 ± 3.3	21.1 ± 3.3	0.594	21.3 ± 4.1	21 ± 2.8	0.839
HR (beats/min)	69.0 ± 8.3	69.0 ± 11.8	0.991	72.9 ± 10	66.3 ± 12.4	0.389
Systolic blood pressure (mmHg)	121.2 ± 9.4	118.8 ± 11.1	0.608	115.7 ± 10.2	120.3 ± 11.5	0.764
Diastolic blood pressure (mmHg)	71.2 ± 8.2	80.5 ± 6.7	0.756	73.6 ± 7.9	71.9 ± 10.3	0.805
History of smoking,* n* (%)	0 (0%)	5 (12%)	0.189	1 (6%)	4 (17%)	0.764
Dyspnea, *n* (%)	0 (0%)	5 (12%)	0.189	1 (6%)	4 (17%)	0.764
Chest pain (atypical), *n* (%)	0 (0%)	3 (7%)	0.301	0 (0%)	3 (7%)	0.764
Peripheral edema, *n* (%)	0 (0%)	4 (10%)	0.193	1 (6%)	3 (7%)	0.805
Palpitations, *n* (%)	0 (0%)	6 (15%)	0.065	2 (12%)	4 (17%)	0.805
Dizziness, *n* (%)	0 (0%)	7 (17%)	0.060	2 (12%)	5 (21%)	0.805
Loss of consciousness, *n* (%)	0 (0%)	2 (5%)	0.494	0 (0%)	2 (8%)	0.805
NYHA class	1 (1–1)	1 (1–1)	> 0.99	1 (1–1)	1 (1–1)	> 0.99
Laboratory values						
Creatinine (μmol/L)	-	65.6 ± 17.7	-	55.7 ± 11.8	73 ± 18	0.032
Urea (mmol/L)	-	5.1 ± 1.4	-	4.4 ± 1.1	5.5 ± 1.4	0.149
AST (U/L)	-	30.0 ± 13.5	-	32.8 ± 15.5	28.0 ± 11.8	0.764
ALT (U/L)	-	34.2 ± 26.8	-	40.9 ± 27.8	29.2 ± 25.5	0.764
ALP (U/L)	-	119.1 ± 74.6	-	131.5 ± 91.4	110.0 ± 59.9	0.764
Total bilirubin (μmol/L)	-	16.9 ± 9.1	-	15.6 ± 7.7	17.9 ± 10.1	0.805
Direct bilirubin (μmol/L)	-	6.5 ± 3.6	-	5.8 ± 2.7	7.0 ± 4.1	0.764
Iron (μmol/L)	-	15.6 ± 7.9	-	16.5 ± 8.9	15.0 ± 7.1	0.805
Ceruloplasmin (mg /L)	-	50.9 ± 31.3	-	58.2 ± 40.3	45.4 ± 22.0	0.764
Copper, serum levels (μmol/L)	-	2.4 ± 1.5	-	2.3 ± 1.4	2.5 ± 1.6	0.805
Zinc, serum levels (μmol/L)	-	15.5 ± 5.7	-	16.1 ± 5.5	15.0 ± 5.9	0.805
Copper, urine levels (μg/dL)	-	26.3 ± 27.0	-	31.2 ± 35.5	22.7 ± 18.5	0.764
Copper, 24-h urine (μg/24 h)	-	623.1 ± 689.5	-	664.7 ± 675.4	592.4 ± 713.2	0.839
Zinc, urine levels (μg/dL)	-	106.9 ± 97.2	-	107.9 ± 65.9	106.2 ± 116.6	> 0.99
Zinc, 24-h urine (μg/24 h)	-	2170.7 ± 2212.8	-	2014.0 ± 1205.8	2286.5 ± 2754.9	0.805
Echocardiographic abnormalities, *n* (%)	-	0 (0%)	-	0 (0%)	0 (0%)	> 0.99
ECG abnormalities, *n* (%)	-	19 (46%)		5 (29%)	14 (58%)	0.371

Continuous variables are presented as mean ± standard deviation, and categorical variables are presented as *n* (%). Differences between groups were calculated using Student’s *t*-test or Fisher's exact test

*BSA* body surface area, *BMI* body mass index, *HR* heart rate, *NYHA class* New York Heart Association class, *AST* aspartate transaminase, *ALT* alanine transaminase, *ALP* alkaline phosphatase, *ECG* electrocardiogram

### Cardiac morphology and function

Cardiac morphological and functional parameters (including LV, RV, and LA) are presented in Table 3. There was no significant difference in cardiac morphological and functional parameters between the WD group and controls, the WD-neuro^+^ group and WD-neuro^−^ group (all *p* > 0.05).

**Table 3:** CMR parameters in WD patients and controls

Parameters	Controls (*n* = 40)	WD (*n* = 41)	*p*	WD-neuro − (*n* = 17)	WD-neuro + (*n* = 24)	*p*
Cardiac morphology and function						
LVEF (%)	60.5 ± 6.0	59.2 ± 5.9	0.652	60.5 ± 6.5	58.3 ± 5.4	0.703
LVCI (L/min/m^2^)	3.1 ± 0.6	3.1 ± 0.8	0.976	3.3 ± 1.0	3.0 ± 0.7	0.703
LVEDVi (mL/m^2^)	74.0 ± 10.2	77.4 ± 11.2	0.477	75.8 ± 11.7	78.4 ± 10.9	0.703
LVESVi (mL/m^2^)	29.2 ± 6	32.2 ± 6.3	0.204	31.1 ± 7.1	32.9 ± 5.8	0.703
LVSVi (mL/m^2^)	45.2 ± 7.8	45.2 ± 9.2	0.978	44.8 ± 10.9	45.5 ± 8.0	0.795
LV mass index (g/m^2^)	42.0 ± 6.9	42.9 ± 6.9	0.786	42.2 ± 6.3	43.5 ± 7.3	0.703
RVEF (%)	61.5 ± 7.7	59.0 ± 9.3	0.654	61.2 ± 8.9	57.4 ± 9.4	0.552
RVCI (L/min/m^2^)	3.2 ± 0.7	3.2 ± 0.9	0.937	3.3 ± 0.9	3.1 ± 1.0	0.664
RVEDVi (mL/m^2^)	76 ± 14.8	78.6 ± 13.5	0.654	74.9 ± 13.7	81.3 ± 13.0	0.552
RVESVi (mL/m^2^)	29.5 ± 8.9	32.3 ± 9.1	0.654	29.1 ± 8.3	34.6 ± 9.2	0.440
RVSVi (mL/m^2^)	46.5 ± 9.7	46.3 ± 10.5	0.937	45.9 ± 10.9	46.6 ± 10.4	0.931
LAEF (%)	59.5 ± 6.7	59.1 ± 6.5	0.937	58.0 ± 6.2	59.8 ± 6.7	0.664
LAVi max (mL/m^2^)	33.6 ± 6.7	35.6 ± 10.1	0.654	35.6 ± 11.4	35.6 ± 9.3	> 0.99
LAVi min (mL/m^2^)	13.7 ± 4.0	14.5 ± 4.7	0.654	15.2 ± 5.9	14.1 ± 3.6	0.679
2D LV-Strain						
GRS (%)	34.0 ± 4.7	32.6 ± 4.9	0.226	33.5 ± 5.2	31.9 ± 4.7	0.672
GCS (%)	-19.4 ± 1.6	-18.9 ± 1.7	0.226	-19.1 ± 1.9	-18.7 ± 1.6	0.715
GLS (%)	-18.1 ± 2.1	-17.4 ± 2.5	0.226	-18.1 ± 3.0	-17.0 ± 2.1	0.600
Tissue characterization						
Global native T1 (ms)	1046.5 ± 33.1	1085.1 ± 39.1	< 0.001	1082.8 ± 37.8	1086.7 ± 40.7	0.795
Prolonged T1, %	5.1	42.1	< 0.001	47.1	38.5	0.703
Global T2 (ms)	51.5 ± 2.6	54.2 ± 3.3	< 0.001	53.8 ± 3.1	54.5 ± 3.6	0.703
Prolonged T2, %	12.5	28.5	0.006	25.0	31.0	0.345
Global ECV (%)	24.3 ± 3.7	31.8 ± 3.6	< 0.001	30.3 ± 2.5	32.9 ± 3.8	0.042
Prolonged ECV, %	4.0	66.5	< 0.001	57.8	72.6	0.042
LGE performed						
Non-ischemic LGE present,* n* (%)	0 (0%)	32 (78%)	0.004	15 (88%)	17 (71%)	0.672
RVIP,* n* (%)	0 (0%)	15 (37%)	0.004	4 (24%)	11 (46%)	0.600
Interventricular septum,* n* (%)	0 (0%)	9 (22%)	0.005	3 (18%)	6 (25%)	> 0.99
Inferior,* n* (%)	0 (0%)	3 (8%)	0.241	1 (4%)	2 (8%)	> 0.99
Inferolateral,* n* (%)	0 (0%)	5 (12%)	0.110	2 (12%)	3 (13%)	> 0.99

Continuous variables are presented as mean ± standard deviation, and categorical variables are presented as *n* (%). Differences between groups were calculated using Student’s *t*-test or Fisher's exact test

*LVEF* left ventricular ejection fraction, *LVEDVi* left ventricular end-diastolic volume index, *LVESVi* left ventricular end-systolic volume index, *LVSVi* left ventricular stroke volume index, *ECV* extracellular volume, *GRS* global radial strain, *GLS* global longitudinal strain, *GCS* global circumferential strain, *LGE* late gadolinium enhancement, *RVIP* right ventricular insertion point

### CMR tissue characterization

**Fig. 2:**
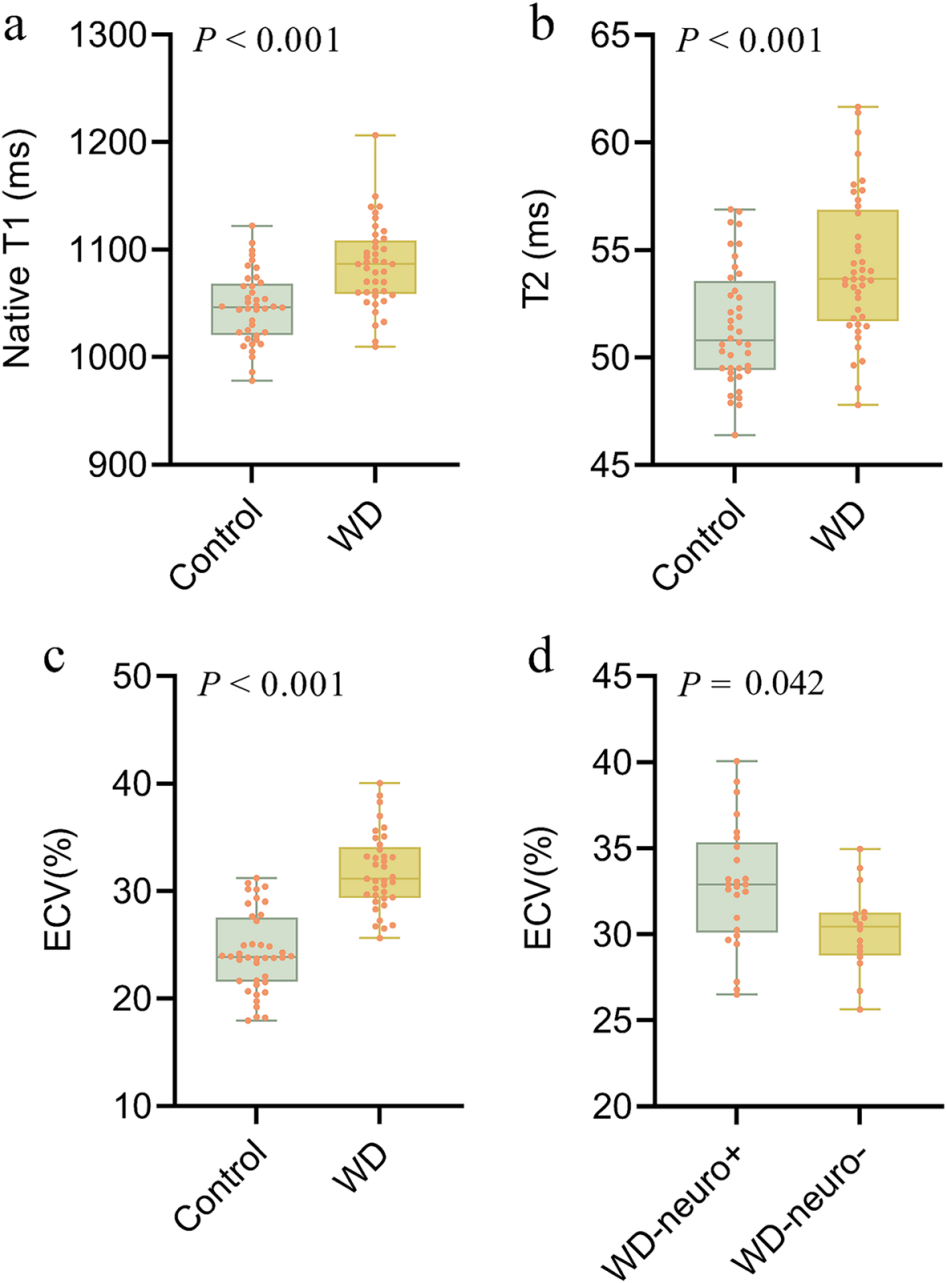
The comparison of nativeT1 (**a**), T2 (**b**), and ECV (**c**) between WD group and controls. The comparison of ECV (**d**) between WD-neuro + and WD-neuro − group. WD Wilson disease, ECV extracellular volume

The global native T1 value of the WD group (1085.1 ± 39.1 ms) was significantly higher than healthy controls (1046.5 ± 33.1 ms) (*p* < 0.001) (Fig. 2). No significant difference was found between the WD-neuro + (1086.7 ± 40.7 ms) and WD-neuro − group (1082.8 ± 37.8 ms) (*p* = 0.795). According to segmental myocardial involvement analysis, 42.1% of the WD group, 38.5% of the WD-neuro + group, and 47.1% of the WD-neuro − group showed prolonged T1 values (Table 3). Segmental analysis of global native T1 WD-neuro + patients and WD-neuro − patients showed that prolonged native T1 values were predominately involved in the interventricular septum and inferior wall (Fig. 4).

The global ECV value of the WD group (31.8 ± 3.6%) was significantly higher than healthy controls (24.3 ± 3.7%) (*p* < 0.001), and the global ECV value of the WD-neuro + group (32.9 ± 3.8%) was significantly higher than that of the WD-neuro − group (30.3 ± 2.5%) (*p* = 0.042) (Fig. 2). According to segmental myocardial involvement analysis, 66.5% of the WD group, 72.6% of the WD-neuro + group, and 57.8% of the WD-neuro − group showed prolonged ECV values. In addition, a greater number of prolonged segments were observed in the WD-neuro + group compared with the WD-neuro − group (*p* = 0.042) (Table 3). Multiple linear regression analysis showed that only the subtype of WD had a significant effect on ECV (*β* =  − 3.50, *p* = 0.012), while the effects of age and gender on ECV did not reach significant levels (*p* = 0.230 and 0.870, respectively).

The T2 value of the WD group (54.2 ± 3.3 ms) was significantly higher than healthy controls (51.5 ± 2.6 ms) (*p* < 0.001) (Fig. 2). No significant difference was found between the WD-neuro + (54.5 ± 3.6 ms) and WD-neuro − group (53.8 ± 3.1 ms) (*p* = 0.703). According to segmental myocardial involvement analysis, 28.5% of the WD group, 31.0% of the WD-neuro + group, and 25.0% of the WD-neuro − group showed prolonged T2 values (Table 3).

### LGE parameters

**Fig. 3:**
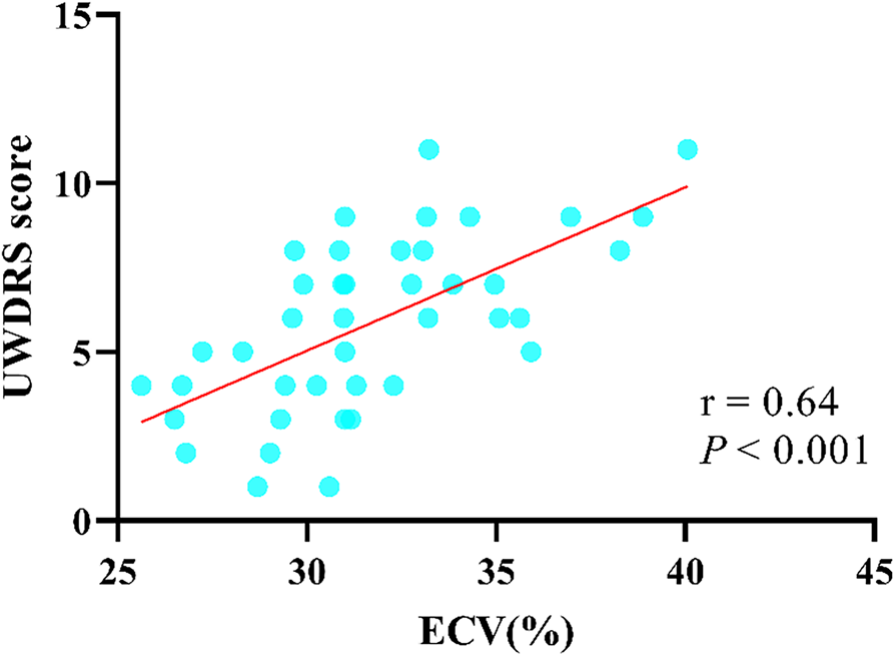
The correlation between UWDRS score and ECV value. Pearson correlation was used to assess linear relationships between variables. UWDRS Unified Wilson Disease Rating Scale, ECV extracellular volume

The presence of LGE and the distribution of LGE-positive segments of WD patients were presented in Table 3. In WD patients, 78% (32/41) of the patients exhibited non-ischemic LGE presence, with 37% (15/41) located in right ventricular insertion point (RVIP), 22% (9/41) in the interventricular septum, 8% (3/41) in the inferior region, and 12% (5/41) in the inferolateral region. However, we did not observe the statistical difference in LGE between the WD-neuro + group and WD-neuro − group. Patients with WD were divided into two groups according to the presence or absence of LGE (LGE positive vs. LGE negative), and it was revealed that T1 and ECV values were not significantly different between the groups (*p* = 0.573, *p* = 0.831, respectively). Correlation analysis was performed in all WD patients. The UWDRS score was significantly correlated with ECV (Pearson’s *r* = 0.64, *p* < 0.001) (Fig. 3).

### Intra- and inter-observer reproducibility

The T1, T2, global radial strain (GRS), global circumferential strain (GCS), and global longitudinal strain (GLS) demonstrated excellent intraobserver and interobserver reproducibility as evidenced by correlation coefficient (ICC) (*r* = 0.99, 0.96, 0.95, 0.94, and 0.97 for intraobserver analyses and *r* = 0.97, 0.92, 0.89, 0.90, and 0.92 for interobserver analyses), respectively (Table 4).

**Table 4:** Intra- and inter-observer variability of native T1, T2, GRS, GCS, and GLS

Parameters	Intra-observer		Inter-observer	
	**ICC**	**95% CI**	**ICC**	**95% CI**
Native T1 (ms)	0.99	0.98–0.99	0.97	0.93–0.99
T2 (ms)	0.96	0.92–0.98	0.92	0.89–0.96
GRS (%)	0.95	0.92–0.98	0.89	0.86–0.94
GCS (%)	0.94	0.92–0.97	0.90	0.86–0.95
GLS (%)	0.97	0.96–0.98	0.92	0.89–0.97

*GRS* Global radial strain, *GLS* Global longitudinal strain, *GCS* Global circumferential strain

## Discussion

To date, this study is the largest Asian population prospective clinical and imaging study evaluating a cardiac manifestation of patients suffering from WD. This study suggested the clinical application value of CMR imaging by detecting the characteristics of myocardial involvement in WD patients. The key findings of this study included: (1) native T1 and T2 and ECV values increased in WD patients without significant left ventricular dysfunction; (2) WD-neuro + patients presented with more severe myocardial damage compared to WD-neuro − patients.

In our study, most of the WD patients had no obvious clinical symptoms, and the ventricular ejection fractions were preserved. Nevertheless, in clinical context, myocardial injury in patients with WD was suspected based on electrocardiogram findings or self-reported symptoms like chest tightness, chest pain, and palpitations. Hence, the significance in utilizing CMR tissue characterization imaging to accurately detect myocardial involvement and understand its specific characteristics.

Previous studies have demonstrated that native T1 can reflect myocardial edema, necrosis, and fibrosis [29, 30]. ECV can further detect the degree of myocardial interstitial fibrosis [22, 31, 32]. Both can be used to quantitatively evaluate diffuse fibrosis [29]. In our study, compared with the controls, the values of native T1 and ECV in WD patients were significantly increased, which was consistent with the studies of Salatzki et al. [6] and Deng et al. [7] In myocardial segmental analysis, 42.1% of native T1 values and 66.5% of ECV values were prolonged. Therefore, we believe that myocardial diffuse fibrosis is one of the characteristics of myocardial damage in WD patients. Moreover, Factor et al. [12] found myocardial fibrosis in autopsies of young WD patients, which further verified the rationality of our conclusion. In addition, we also observed that the T2 value of WD patients was significantly increased compared with the controls, and 59.5% of T2 values were prolonged in myocardial segmental analysis. Elevated transverse relaxation time (T2) is specific for increased myocardial water content, increased free water, and is used as an index of myocardial edema [33]. Therefore, we infer that potential myocardial edema may also be one of the characteristics of myocardial damage in patients with WD. However, Salatzki et al. [6] did not reveal a noteworthy distinction in T2 values between the WD group and control group. This finding could potentially be attributed to variations in factors such as a smaller number of short-axis slices or differences in ethnicity and sample size.

**Fig. 4:**
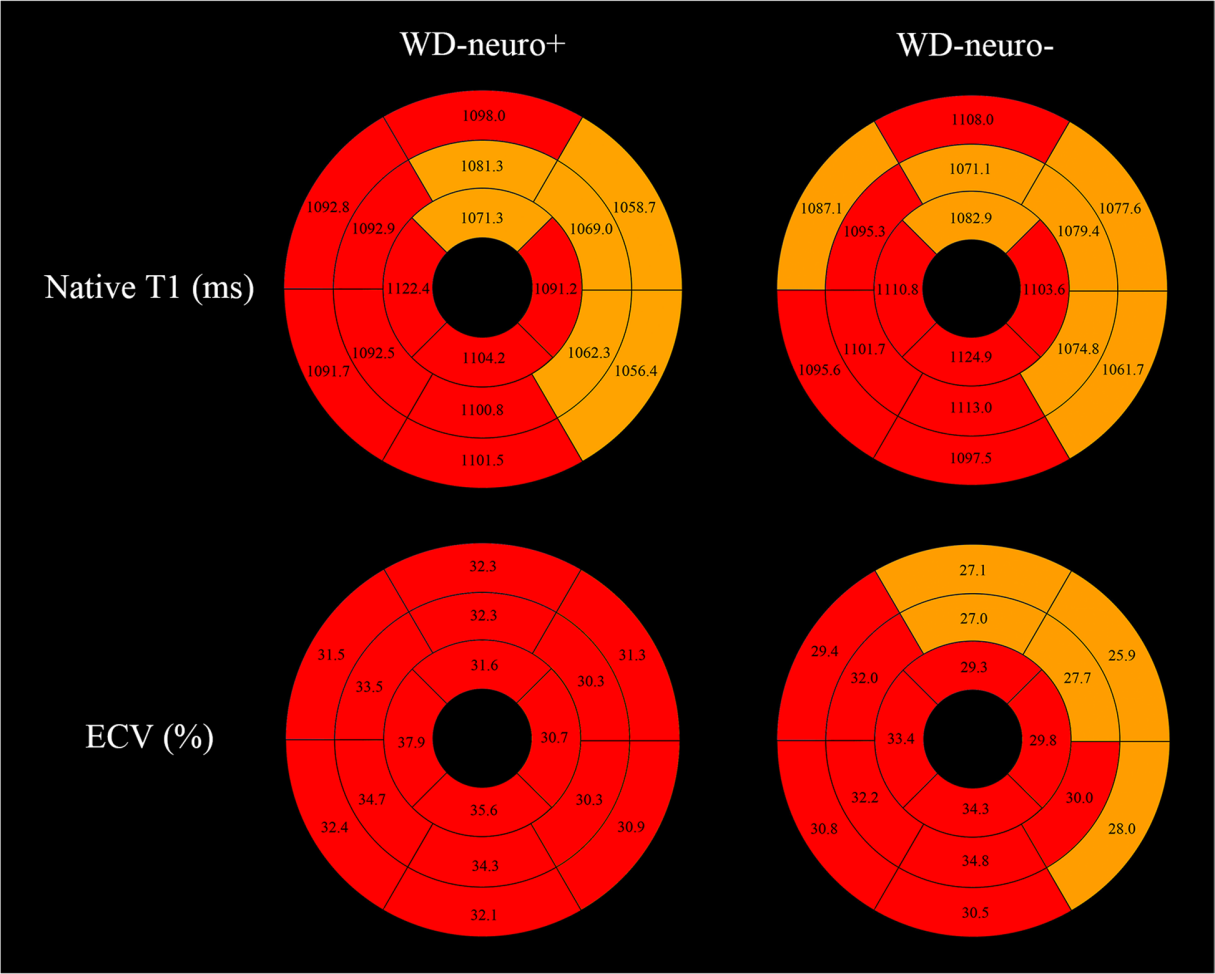
Bull’s eye plots comparing the global native T1 and the ECV values of WD-neuro + and WD-neuro − group. Segmentation was performed according to the AHA 16-segment model in three short-axis slices. The native T1 and ECV values of more than the cut-off 1090 ms and 29% are shown with red color. AHA American Heart Association, ECV extracellular volume

**Fig. 5:**
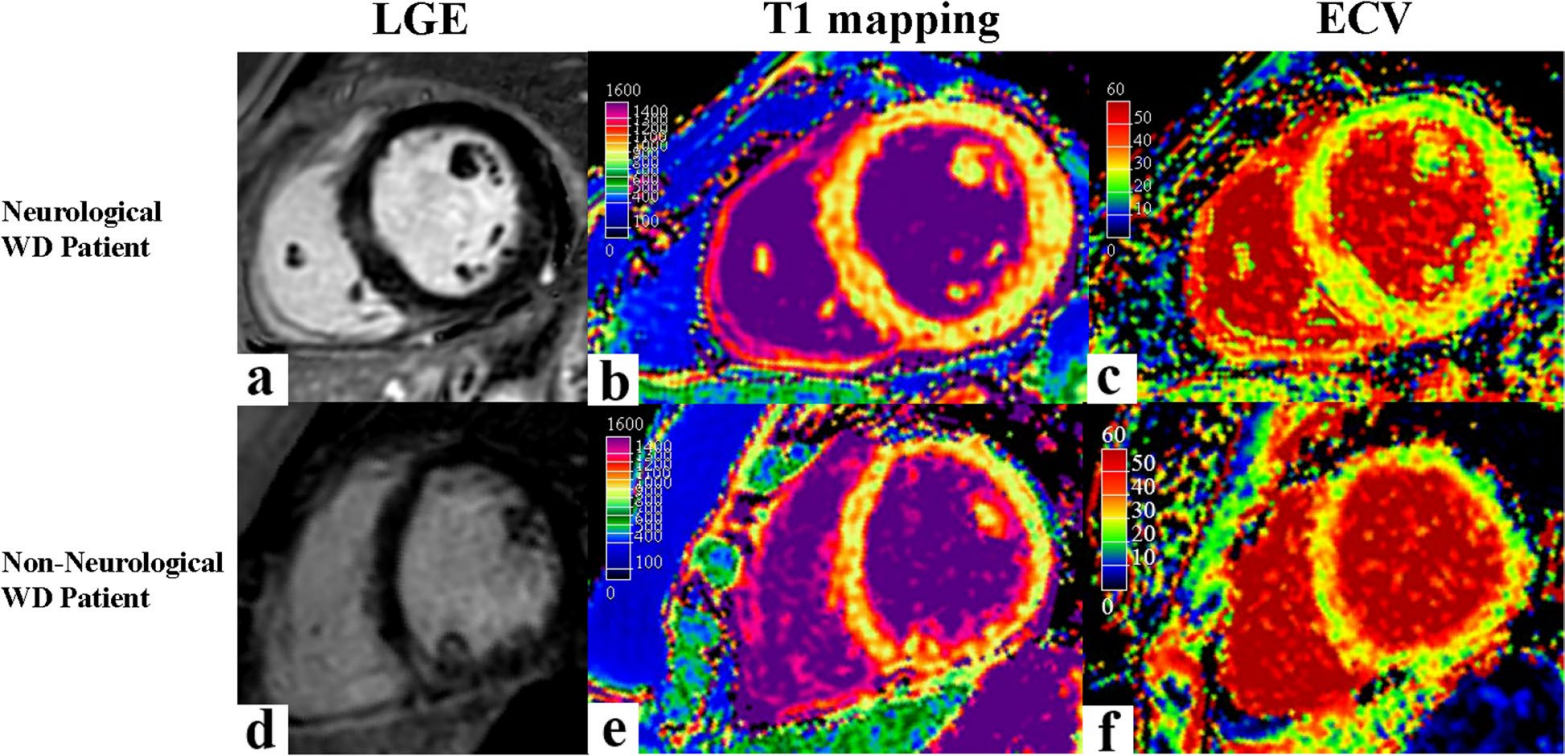
A 31-year-old male neurological WD patient showed late gadolinium enhancement in the septal and RVIP regions indicated by the white arrow (**a**), increased T1 value (1100.2 ms) (**b**), and elevated ECV value (31.8%) (**c**). A 25-year-old female non-neurological WD patient showed late gadolinium enhancement in the RVIP indicated by the white arrow (**d**), increased T1 value (1082.7 ms) (**e**), and elevated ECV value (30.3%) (**f**)

In our study, there was a significant difference in ECV between the WD-neuro + group and WD-neuro − group, and the same results also were found in myocardial segmental analysis (Figs. 4 and 5). To our knowledge, this is the first report delineating variations in myocardial involvement between WD-neuro + and WD-neuro − patients in Asia. These findings align with prior work by Salatzki et al. [6]. The more severe myocardial damage observed in WD-neuro + patients compared to WD-neuro − patients can be attributed to the longer duration of copper overload in those with neurological manifestations. WD-neuro + patients are considered to be at a more advanced disease stage, as the emergence of neurological symptoms typically occurs later in the disease course [34, 35]. The subtle initial signs of WD often delay clinical diagnosis and treatment. Consequently, by the time neurological symptoms appear, WD-neuro + patients have likely been exposed to elevated copper levels for an extended period compared to WD-neuro − patients. The prolonged copper overload supports the direct cardiotoxic effects of copper on myocardial tissues in WD, though the specific mechanisms require further investigation.

LGE-MRI is the reference standard for the detection of myocardial fibrosis and has been shown to have excellent sensitivity and specificity in the detection of myocardial scar tissue [36, 37]. Previous studies have shown that myocardial LGE is associated with prognosis and the incidence of adverse events [38, 39]. In our study, 78% (32/41) of the patients showed positive LGE. The location of LGE mainly appeared in RVIP (15/41) and interventricular septum (9/41) and a few appeared in the inferior wall (3/41) and inferolateral wall (5/41). The LGE pattern and incidence of WD patients found in our study were similar to the study of Salatzki et al. [6], but the presence of LGE in RVIP was much lower than that in the study by Quick et al. [13] (15/41 vs. 58/61). This difference could be clarified by noting that, in contrast to Quick’s study, the participants in our study were younger (28 years vs. 44 years) and experienced a shorter duration of copper exposure, potentially resulting in mild cardiac damage. In addition, a recent case report illustrating sudden cardiac death associated with LGE in a patients with Wilson disease underscores the significance of fibrosis as a potential significant indicator of WD [40].

Interestingly, there was no significant difference between LGE negative group and LGE positive group in native T1 and ECV values. This lack of discrepancy can be attributed to fundamental disparities between LGE and quantitative mapping techniques. LGE accentuates focal myocardial fibrosis based on regional differences in gadolinium uptake, yet its utility in detecting diffuse interstitial expansion is restricted. Conversely, native T1 and ECV relaxation times and ECV delineate global myocardial fibrosis burden irrespective of regional involvement [29, 41]. Consequently, diffuse extracellular matrix expansion in LGE-negative segments may yield native T1 and ECV measures comparable to those obtained in LGE-positive regions exhibiting concomitant focal scarring.

In our study, no significant difference was observed in 2D LV-Strain (GRS, GCS, GLS) between the WD and control groups, which was inconsistent with previous studies [6, 8]. In the studies of Salatzki et al. [6] and Zhang et al. [8], LV-GRS and LV-GCS in WD patients were significantly lower than those in controls. This difference may be due to the younger patients in our study and the shorter duration of copper exposure.

The UWDRS score was positively correlated with ECV values in this study. The same findings were found in the study of Salatzki et al. [6]. It showed that the severity of neurological symptoms is related to the degree of myocardial fibrosis. But we did not find a correlation between the UWDRS score and cardiac function or other CMR tissue characteristic parameters. Therefore, whether the UWDRS score is related to the degree of cardiac involvement needs to be verified in a larger cohort and pathological examination.

The findings of this study have significant clinical implications for the management of WD. CMR enabled early detection of myocardial involvement in WD patients, even in those with normal cardiac function and subtle or absent clinical symptoms. This early identification is profoundly clinically relevant as it permits risk stratification and identification of patients at higher risk of cardiac complications. Moreover, the differences in myocardial involvement between WD patients with and without neurological symptoms highlights the heterogeneity of the disease. CMR could facilitate personalized treatment by revealing the extent of myocardial damage in individual patients. Additionally, longitudinal CMR follow-up allows dynamic monitoring of cardiovascular disease progression, informing treatment adjustments and evaluating therapeutic efficacy. While providing valuable insights, this study underscores the need for further research on cardiac involvement in WD. Future investigations should aim to validate these findings in larger cohorts, explore the potential of CMR-guided interventions, and elucidate the mechanisms underlying copper-induced myocardial damage.

### Study limitations

Firstly, our study had a limited sample size, potentially introducing biases. Secondly, our study participants were younger. It is possible that older patients may exhibit more severe myocardial impairment, which warrants further study. Thirdly, no biopsies of the myocardium prevented us from establishing a direct histological correlation between regions of elevated native T1, ECV, and LGE in the myocardium.

## Conclusions

In conclusion, CMR can detect myocardial injury in WD patients prior to the onset of cardiac functional impairments. Additionally, the nature of myocardial involvement exhibited distinction between WD-neuro + patients and WD-neuro − patients. Myocardial engagement is more pronounced in WD-neuro + patients.

## Data Availability

The original contributions presented in the study are included in the article. Further inquiries can be directed to the corresponding authors.

## Abbreviations

CMR: Cardiac magnetic resonance
ECV: Extracellular volume fraction
GCS: Global circumferential strain
GLS: Global longitudinal strain
GRS: Global radial strain
LGE: Late gadolinium enhancement
LV: Left ventricle
RV: Right ventricle
RVIP: Right ventricular insertion point
UWDRS: Unified Wilson Disease Rating Scale
WD: Wilson disease
WD-neuro-: Patients with a primarily hepatic involvement
WD-neuro +: Patients with at least one neurological symptom
